# Effect of Empagliflozin on Cardiovascular Mortality and Heart Failure Hospitalizations: A Systematic Review and Meta-Analysis of Randomized Controlled Trials

**DOI:** 10.7759/cureus.85669

**Published:** 2025-06-09

**Authors:** Uzma Rauf, Farrukh Ansar, Muhammad Shazib Ali, Fizza Chudhary, Muhammad Haris Zafar, Abdullah Azzam, Muhammad Adeel Amjad, Mehak Qmar Siddique, Mohammad S Rauf, Muhammad Bilal Ahmad

**Affiliations:** 1 Medicine, St. Vincent Medical Center, Toledo, USA; 2 Medicine, Alkhidmat Raazi Hospital, Rawalpindi, PAK; 3 Orthopedics, Ihsan Mumtaz Hospital, Lahore, PAK; 4 Pharmacology and Therapeutics, Niazi Medical and Dental College, Sargodha, PAK; 5 Anatomy, Amna Inayat Medical and Educational Complex, Sheikhupura, PAK; 6 Medicine and Surgery, Northwest General Hospital, Peshawar, PAK; 7 Medicine, Quaid-e-Azam International Hospital, Islamabad, PAK

**Keywords:** cardiovascular mortality, empagliflozin, heart failure, heart failure hospitalizations, sodium-glucose co-transporter-2 (sglt2) inhibitors

## Abstract

Empagliflozin, a sodium-glucose cotransporter 2 (SGLT2) inhibitor, has demonstrated clinically meaningful benefits in patients with heart failure (HF). This systematic review and meta-analysis aimed to assess the effect of empagliflozin on cardiovascular mortality and hospitalization for heart failure (HHF) across randomized controlled trials (RCTs). A comprehensive literature search was conducted across PubMed, Embase, Scopus, and Google Scholar, identifying five eligible RCTs. Studies were included if they evaluated empagliflozin's impact on cardiovascular mortality and HHF. A meta-analysis was performed using hazard ratios (HRs) for cardiovascular death and HHF. Data were synthesized using fixed- or random-effects models based on the degree of heterogeneity. The meta-analysis included 23,344 patients, of whom 12,849 received empagliflozin. Cardiovascular mortality was significantly reduced with empagliflozin (HR 0.86; 95% CI: 0.78-0.96). For HHF, empagliflozin reduced the risk by 30% (HR 0.70; 95% CI: 0.64-0.77) across all trials. Sensitivity analysis confirmed the robustness of these results. Empagliflozin significantly reduces cardiovascular mortality and HHF, supporting its use as a cornerstone therapy in heart failure management, especially in patients at high cardiovascular risk. These findings highlight the potential of empagliflozin as a key therapeutic intervention in heart failure care.

## Introduction and background

Heart failure (HF) is a major global health burden, affecting over 64 million people and contributing significantly to morbidity, mortality, and healthcare costs [1]. It remains a leading cause of hospital admissions and is associated with high rates of rehospitalization and early mortality [1]. Despite therapeutic advances, HF prognosis remains poor, especially in patients with recurrent hospitalizations or comorbidities like diabetes and chronic kidney disease [2]. Cardiovascular mortality and hospitalization for heart failure (HHF) are key clinical endpoints in determining quality of life, resource utilization, and long-term survival in patients with HF [3]. Identifying treatments that can favorably influence these outcomes is of paramount clinical importance.

Sodium-glucose cotransporter-2 (SGLT2) inhibitors, originally developed as antihyperglycemic agents, have demonstrated significant cardiovascular and renal benefits that extend beyond glucose lowering [4]. Empagliflozin has emerged as a leading agent within this class as a leading therapeutic agent, with pivotal trials demonstrating its potential to modify the clinical trajectory of cardiovascular disease [5]. Empagliflozin is an SGLT2 inhibitor initially approved for glycemic control in patients with type 2 diabetes [5]. The landmark Empagliflozin Cardiovascular Outcome Event Trial in Type 2 Diabetes Mellitus Patients (EMPA-REG OUTCOME) trial was the first to demonstrate a significant reduction in cardiovascular mortality and HHF in patients with type 2 diabetes and established atherosclerotic cardiovascular disease [6]. This was a turning point in the understanding of SGLT2 inhibitors, shifting them from glucose-centric therapies to cardiorenal agents with broad systemic effects.

Subsequent trials, Empagliflozin Outcome Trial in Patients With Chronic Heart Failure and a Reduced Ejection Fraction (EMPEROR-Reduced) [7] and Empagliflozin Outcome Trial in Patients With Chronic Heart Failure With Preserved Ejection Fraction (EMPEROR-Preserved) [8], expanded the scope of empagliflozin’s benefits to include patients with heart failure with reduced and preserved ejection fraction (HFrEF and HFpEF, respectively), irrespective of diabetic status [7,8]. Moreover, mechanistic studies have revealed that empagliflozin may exert its effects through natriuresis, plasma volume reduction, improved myocardial energetics, and anti-inflammatory pathways, supporting its utility even in non-diabetic patients [9]. While these mechanisms are biologically plausible, they are primarily derived from small-scale mechanistic studies and should be interpreted as supportive rather than definitive clinical evidence [9]. However, findings from the recent Empagliflozin in Patients With Acute Myocardial Infarction (EMPACT-MI) trial did not show statistically significant reductions in mortality, highlighting population-specific variability [10]. Nevertheless, due to its relevance to the outcomes of interest (CV mortality and HHF), it is included to present a comprehensive evidence base and to avoid selective outcome reporting. Empagliflozin has also been associated with reductions in LV end-diastolic volume, suggesting reverse remodeling [11].

Although multiple individual trials have evaluated the role of empagliflozin in different cardiovascular contexts, a focused synthesis addressing its dual impact on cardiovascular mortality and HHF across randomized controlled trials (RCTs) is warranted. However, prior meta-analyses have typically assessed these outcomes separately or focused on narrower populations. Our review uniquely synthesizes both cardiovascular mortality and HHF as co-primary endpoints, providing a more integrated understanding of empagliflozin’s clinical benefits across a spectrum of cardiovascular risk profiles. A synthesis across diabetic and non-diabetic populations and both HFrEF and HFpEF phenotypes may inform clinical decision-making. We acknowledge that differences in patient populations, including variations in HF phenotype and the inclusion of post-MI patients in EMPACT-MI, may introduce heterogeneity. Such diversity reflects real-world populations and should be addressed through sensitivity analysis and transparent discussion in the interpretation of findings.

The objective of this systematic review and meta-analysis is to evaluate the effect of empagliflozin on cardiovascular mortality and HHF by analyzing data from contemporary RCTs. By synthesizing evidence across diverse patient populations, this review aims to clarify the scope and consistency of empagliflozin’s cardiovascular benefits and identify directions for future research and clinical application. Cardiovascular mortality and hospitalization for HHF are selected as co-primary outcomes due to their strong association with poor prognosis, high healthcare utilization, and reduced quality of life in HF patients. Focusing on these endpoints provides clinically meaningful insights into how empagliflozin may alter the disease trajectory. Therefore, this review aims to synthesize current evidence on the cardiovascular efficacy of empagliflozin in diverse HF populations.

## Review

Methodology

Search Strategy

A comprehensive literature search was performed in PubMed, Embase, Scopus, and Google Scholar from database inception to March 2025. The search strategy incorporated Medical Subject Headings (MeSH) and Boolean operators (AND, OR) to enhance both sensitivity and specificity. The complete PubMed MeSH-based query was: “("Empagliflozin"[MeSH Terms] OR "empagliflozin"[All Fields]) AND ("Heart Failure"[MeSH Terms] OR "heart failure"[All Fields] OR "cardiac failure"[All Fields]) AND ("Hospitalization"[MeSH Terms] OR "hospitalization"[All Fields] OR "admission"[All Fields]) AND ("Cardiovascular Diseases/mortality"[MeSH Terms] OR "cardiovascular death"[All Fields]) AND ("Randomized Controlled Trial"[Publication Type] OR "RCT"[All Fields])”. Google Scholar was used primarily for identifying grey literature and additional studies not indexed in other databases.

Reference lists of included trials and relevant systematic reviews were also manually screened for additional eligible studies. The review was conducted in accordance with the PRISMA 2020 (Preferred Reporting Items for Systematic Reviews and Meta-Analyses) guidelines.

Eligibility Criteria

We included RCTs that evaluated the effect of empagliflozin on cardiovascular mortality and HHF in adult patients with or at risk of cardiovascular disease. Studies were eligible if they reported at least one of the primary outcomes and were published in English-language peer-reviewed journals. Trials involving animal models, observational designs, or those lacking outcome-specific data were excluded. Included trials enrolled adult patients with diagnosed heart failure, post-myocardial infarction status, or at high risk of cardiovascular events. Asymptomatic patients without diagnosed cardiovascular disease were excluded.

Study Selection

To enhance objectivity and methodological rigor, two independent teams, each comprising three reviewers, screened all retrieved records. Titles and abstracts were independently evaluated for eligibility, followed by full-text review. After independent team-level screening, results were compared and reconciled between the teams. Manual screening of reference lists was conducted independently by two reviewers to identify additional eligible studies. Discrepancies were resolved through discussion; the principal author adjudicated disagreements. Only trials meeting all inclusion criteria and reporting cardiovascular death and/or HHF outcomes were retained.

Data Extraction and Management

Data extraction was performed independently by both teams using a predesigned standardized template. Extracted variables included study identifiers (first author, publication year), geographic setting, population characteristics, intervention/control details setting, population characteristics, sample size, follow-up duration, outcomes (cardiovascular death and HHF), hazard ratios (HRs) with 95% confidence intervals, adverse events, and risk of bias information. After completion, the extracted data were reconciled and verified by the principal author for consistency. The principal author independently rechecked original study reports and source data to verify accuracy beyond team comparisons. Risk of bias was assessed independently by two reviewers using the Cochrane Risk of Bias 2.0 tool (Cochrane, London, UK). Discrepancies were resolved by consensus or adjudication by the principal author.

Outcomes and Definitions

Primary outcomes: Cardiovascular mortality, defined as death due to cardiovascular causes as adjudicated or reported by study investigators.

HHF definition: Unplanned inpatient admission for worsening heart failure symptoms or decompensation.

Data Synthesis and Statistical Analysis

Meta-analyses were conducted using the inverse-variance method to pool HRs and corresponding 95% confidence intervals. For outcomes with low statistical heterogeneity (I² < 25%), a fixed-effect model was applied; for outcomes with substantial heterogeneity (I² > 50%), a random-effects model (DerSimonian and Laird) was used. For moderate heterogeneity (I² between 25% and 50%), a random-effects model was applied to account for potential variability. Statistical heterogeneity was assessed using the Cochran Q test and I² statistic. Forest plots were used to visualize individual study estimates and the overall pooled effect. 

Sensitivity Analysis

A sensitivity analysis was conducted to evaluate the effect of including the Effects of Empagliflozin on Cardiac Structure, Function, and Clinical Status in Patients With Heart Failure With Reduced Ejection Fraction Without Diabetes (EMPA-TROPISM) trial, which had zero events in the empagliflozin arm. A continuity correction was applied to enable estimation of the HR. The pooled estimates with and without this study were compared to assess the robustness of the results. However, we acknowledge that inclusion of trials with zero events may introduce bias in HR-based meta-analyses, and results should be interpreted cautiously.

Results

Searching Databases

A total of 242 records were identified through database searches (PubMed, Embase, Google Scholar, and Scopus). After removing 124 duplicates, 118 unique records were screened by title and abstract, of which 63 were excluded. The remaining 55 full-text articles were retrieved and assessed for eligibility.

**Figure 1:**
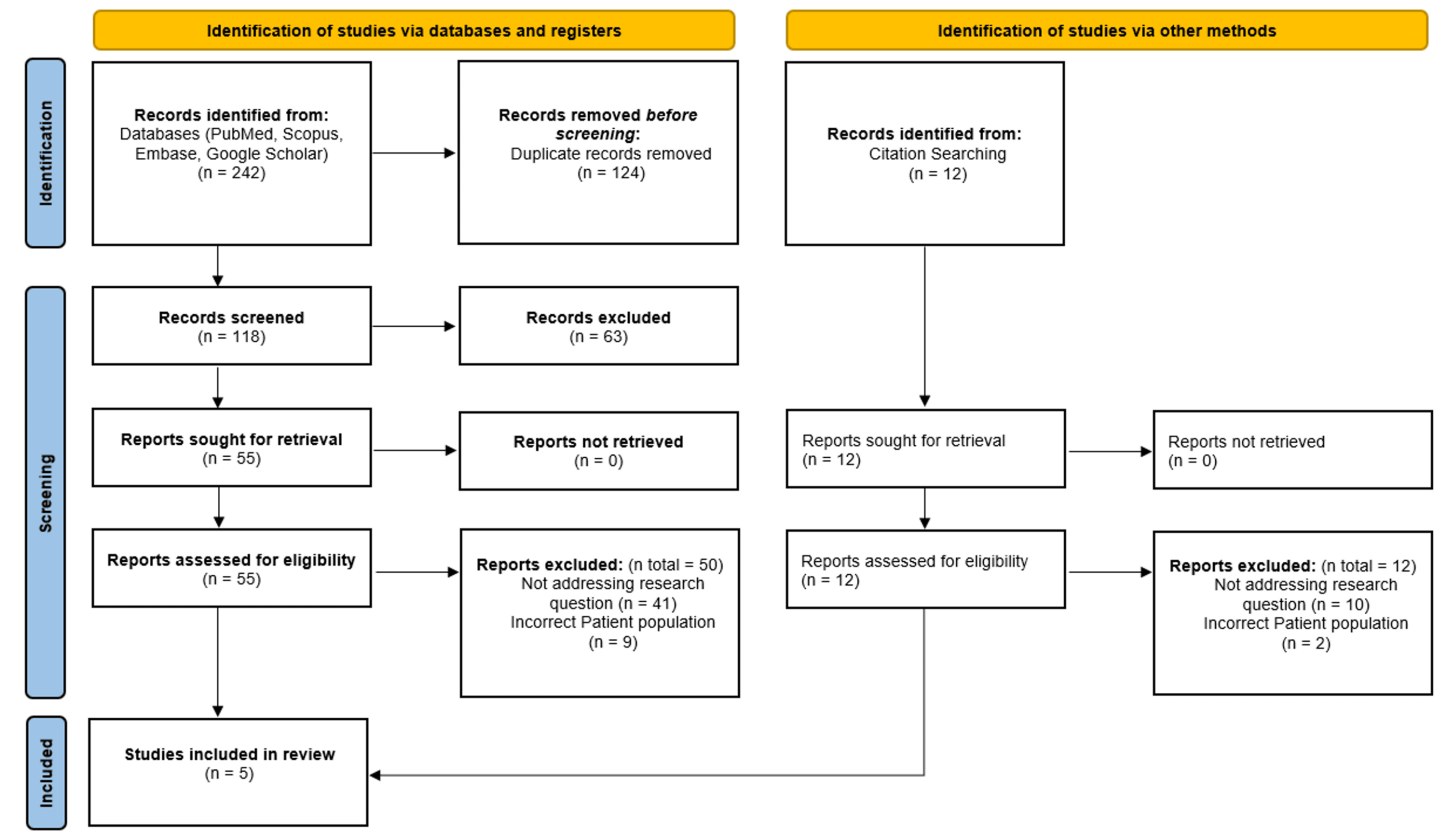
Flowchart of study selection process

Additionally, 12 articles were identified through citation searching and were also assessed in full. This yielded a total of 67 full-text articles assessed for eligibility. After applying inclusion and exclusion criteria, 62 articles were excluded, primarily because they did not address the research question (n=51) or included an incorrect patient population (n=11). Ultimately, five RCTs were included in the final review (Figure 1).

Study Characteristics

This systematic review and meta-analysis included five RCTs assessing the effects of empagliflozin on cardiovascular mortality and HHF. The combined sample comprised 23,344 patients, with 12,849 receiving empagliflozin and 10,495 assigned to placebo. The included trials were EMPEROR-Reduced (n=3,730), EMPA-TROPISM (n=84), EMPA-REG OUTCOME (n=7,020), EMPEROR-Preserved (n=5,988), and EMPACT-MI (n=6,522). Participants represented a broad clinical spectrum, including patients with heart failure with reduced ejection fraction (HFrEF), preserved ejection fraction (HFpEF), type 2 diabetes with high cardiovascular risk, and a post-myocardial infarction setting. Diabetes status varied across trials: EMPA-REG OUTCOME exclusively enrolled patients with type 2 diabetes, EMPA-TROPISM included only non-diabetic participants, and the remaining trials enrolled mixed populations. Table 1 shows the baseline characteristics of the included studies.

**Table 1:** Baseline characteristics of included studies HFrEF: heart failure with reduced ejection fraction; HFpEF: heart failure with preserved ejection fraction; CV: cardiovascular; MI: myocardial infarction; NYHA: New York Heart Association; EMPEROR-Reduced: Empagliflozin Outcome Trial in Patients With Chronic Heart Failure and a Reduced Ejection Fraction; EMPA-TROPISM: Effects of Empagliflozin on Cardiac Structure, Function, and Clinical Status in Patients With Heart Failure With Reduced Ejection Fraction Without Diabetes; EMPA-REG OUTCOME: Empagliflozin Cardiovascular Outcome Event Trial in Type 2 Diabetes Mellitus Patients; EMPEROR-Preserved: Empagliflozin Outcome Trial in Patients With Chronic Heart Failure With Preserved Ejection Fraction; EMPACT-MI: Empagliflozin in Patients With Acute Myocardial Infarction

Study	First Author	Year	Population	Total Sample	Empagliflozin	Placebo
EMPEROR-Reduced [7]	Milton Packer	2020	Adults with chronic HFrEF (NYHA class II–IV, LVEF ≤40%)	3730	1863	1867
EMPA-TROPISM [11]	Carlos G. Santos-Gallego	2021	Non-diabetic patients with HFrEF (NYHA II–III, LVEF <50%)	84	42	42
EMPA-REG OUTCOME [6]	Bernard Zinman	2015	Adults with type 2 diabetes and established CV disease	7020	4687	2333
EMPEROR-Preserved [8]	S.D. Anker	2021	Adults with chronic HFpEF (LVEF >40%, NYHA II–IV), with/without diabetes	5988	2997	2991
EMPACT-MI [10]	Javed Butler	2024	Patients post-acute MI with LV dysfunction (EF <45%) or signs/symptoms of congestion; excluded prior HF	6522	3260	3262

Quality Assessment of the Studies

Risk of bias assessments were independently conducted by two reviewers using the Cochrane RoB 2.0 tool. Discrepancies were resolved by consensus or by consulting a third reviewer. Four trials (EMPEROR-Reduced, EMPA-TROPISM, EMPA-REG OUTCOME, and EMPEROR-Preserved) were judged to be at low risk of bias across all five domains. One study, EMPACT-MI, was rated as having a moderate risk of bias, primarily due to limited blinding and partial reliance on investigator-reported endpoints. No study was excluded based on risk of bias, and overall, the methodological quality was sufficient to support the meta-analytic findings. Details are shown in Table 2.

**Table 2:** Risk of bias assessment using Cochrane RoB 2.0 tool EMPEROR-Reduced: Empagliflozin Outcome Trial in Patients With Chronic Heart Failure and a Reduced Ejection Fraction; EMPA-TROPISM: Effects of Empagliflozin on Cardiac Structure, Function, and Clinical Status in Patients With Heart Failure With Reduced Ejection Fraction Without Diabetes; EMPA-REG OUTCOME: Empagliflozin Cardiovascular Outcome Event Trial in Type 2 Diabetes Mellitus Patients; EMPEROR-Preserved: Empagliflozin Outcome Trial in Patients With Chronic Heart Failure With Preserved Ejection Fraction; EMPACT-MI: Empagliflozin in Patients With Acute Myocardial Infarction

Study	D1: Randomization Process	D2: Deviations From Intended Interventions	D3: Missing Outcome Data	D4: Measurement of Outcome	D5: Selection of Reported Results	Overall Risk of Bias
EMPEROR-Reduced [7]	Low risk	Low risk	Low risk	Low risk	Low risk	Low risk
EMPA-TROPISM [11]	Low risk	Low risk	Low risk	Low risk	Low risk	Low risk
EMPA-REG OUTCOME [6]	Low risk	Low risk	Low risk	Low risk	Low risk	Low risk
EMPEROR-Preserved [8]	Low risk	Low risk	Low risk	Low risk	Low risk	Low risk
EMPACT-MI [10]	Low risk	Some concerns	Low risk	Some concerns	Low risk	Moderate risk

For the primary meta-analysis, we included four studies with estimable HRs and excluded EMPA-TROPISM due to zero events in the treatment group. A separate sensitivity analysis was performed to include EMPA-TROPISM using continuity correction.

Cardiovascular Mortality

Cardiovascular death occurred in 9.98% (186/1863) of empagliflozin-treated patients versus 10.82% (202/1867) in placebo in the EMPEROR-Reduced trial (HR: 0.92; 95% CI: 0.75-1.12). The EMPA-REG OUTCOME trial demonstrated a significant reduction in cardiovascular death (3.7% vs. 5.9%; HR: 0.62; 95% CI: 0.49-0.77; p<0.001), while EMPEROR-Preserved (7.3% vs. 8.2%; HR: 0.91; 95% CI: 0.76-1.09) and EMPACT-MI (4.0% vs. 4.0%; HR: 1.03; 95% CI: 0.81-1.31) showed non-significant differences. In EMPA-TROPISM, no cardiovascular deaths occurred in the empagliflozin group compared to 2.38% (1/42) in the placebo group. Due to the absence of events in the empagliflozin arm, formal HR estimation was not possible; however, the study was included in sensitivity analyses for meta-analytic purposes.

**Figure 2:**
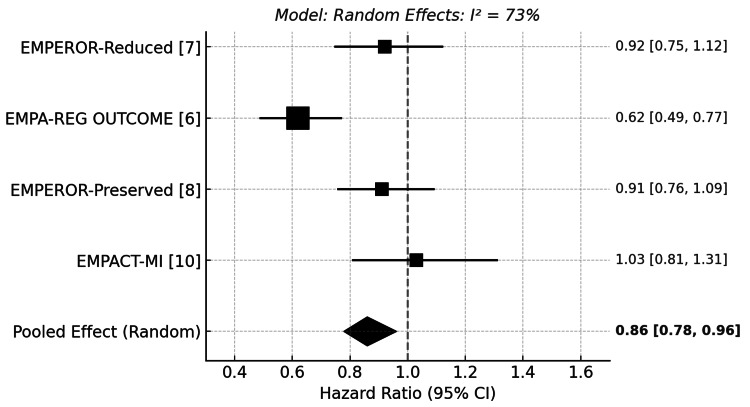
Forest plot for cardiovascular death [6,7,8,10] EMPEROR-Reduced: Empagliflozin Outcome Trial in Patients With Chronic Heart Failure and a Reduced Ejection Fraction; EMPA-REG OUTCOME: Empagliflozin Cardiovascular Outcome Event Trial in Type 2 Diabetes Mellitus Patients; EMPEROR-Preserved: Empagliflozin Outcome Trial in Patients With Chronic Heart Failure With Preserved Ejection Fraction; EMPACT-MI: Empagliflozin in Patients With Acute Myocardial Infarction

In the primary meta-analysis of four trials, empagliflozin was associated with a statistically significant reduction in cardiovascular mortality. The pooled HR was 0.86 (95% CI: 0.78 to 0.96), indicating a 14% relative risk reduction. However, heterogeneity among studies was substantial (I²=73%), and therefore, a random-effects model was applied. (Figure 2) The effect was primarily driven by the EMPA-REG OUTCOME trial, which reported a significant reduction in cardiovascular deaths among patients with type 2 diabetes and high cardiovascular risk. Other trials, including EMPEROR-Reduced, EMPEROR-Preserved, and EMPACT-MI, showed trends in the same direction but did not reach statistical significance individually.

Hospitalization for Heart Failure

The benefit of empagliflozin in reducing HHF was consistently demonstrated across all five studies. In EMPEROR-Reduced, HHF occurred in 10.03% of patients in the empagliflozin arm versus 14.25% in the placebo (HR: 0.69; 95% CI: 0.59-0.81; p<0.001). EMPA-REG OUTCOME reported rates of 2.7% vs. 4.1% (HR: 0.65; 95% CI: 0.50-0.85; p=0.002). EMPEROR-Preserved confirmed this benefit with HHF rates of 8.6% vs. 11.8% (HR: 0.71; 95% CI: 0.60-0.83; p<0.001). EMPACT-MI showed similar reductions (3.6% vs. 4.7%; HR: 0.77; 95% CI: 0.60-0.98). Notably, EMPA-TROPISM reported no HHF events in the empagliflozin group compared to 4.76% (2/42) in placebo, supporting a protective trend.

**Figure 3:**
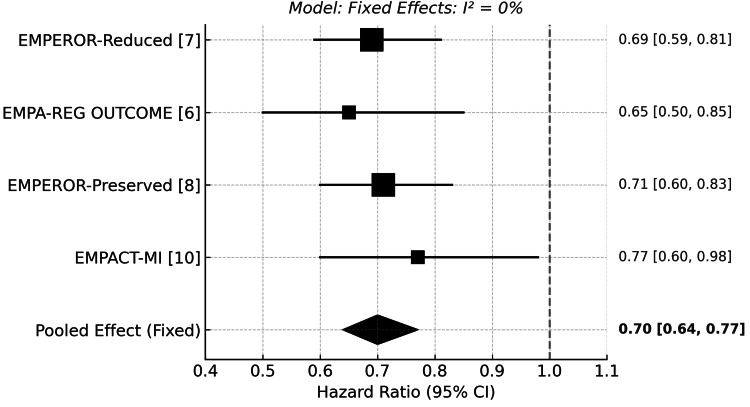
Forest plot for hospitalization for heart failure [6,7,8,10] EMPEROR-Reduced: Empagliflozin Outcome Trial in Patients With Chronic Heart Failure and a Reduced Ejection Fraction; EMPA-REG OUTCOME: Empagliflozin Cardiovascular Outcome Event Trial in Type 2 Diabetes Mellitus Patients; EMPEROR-Preserved: Empagliflozin Outcome Trial in Patients With Chronic Heart Failure With Preserved Ejection Fraction; EMPACT-MI: Empagliflozin in Patients With Acute Myocardial Infarction

Empagliflozin consistently reduced the risk of HFF across all four included trials. The pooled HR was 0.70 (95% CI: 0.64 to 0.77), indicating a 30% relative risk reduction. No heterogeneity was observed (I²=0%), supporting the use of a fixed-effect model. (Figure 3) These findings were remarkably consistent regardless of heart failure phenotype, diabetic status, or baseline cardiovascular risk.

Sensitivity Analysis Including EMPA-TROPISM

**Figure 4:**
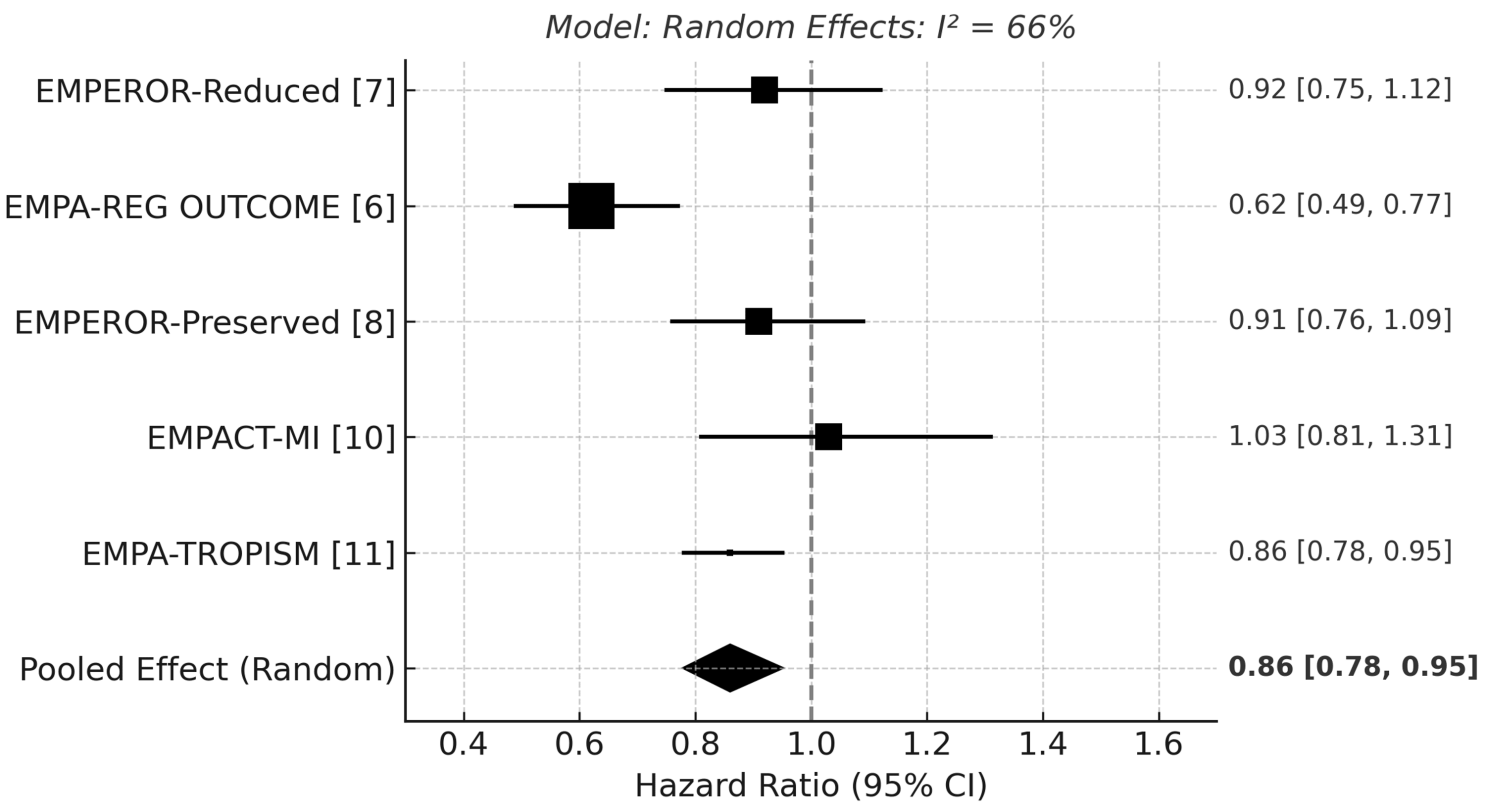
Forest plot for cardiovascular death (sensitivity analysis) [6,7,8,10,11] EMPEROR-Reduced: Empagliflozin Outcome Trial in Patients With Chronic Heart Failure and a Reduced Ejection Fraction; EMPA-TROPISM: Effects of Empagliflozin on Cardiac Structure, Function, and Clinical Status in Patients With Heart Failure With Reduced Ejection Fraction Without Diabetes; EMPA-REG OUTCOME: Empagliflozin Cardiovascular Outcome Event Trial in Type 2 Diabetes Mellitus Patients; EMPEROR-Preserved: Empagliflozin Outcome Trial in Patients With Chronic Heart Failure With Preserved Ejection Fraction; EMPACT-MI: Empagliflozin in Patients With Acute Myocardial Infarction

**Figure 5:**
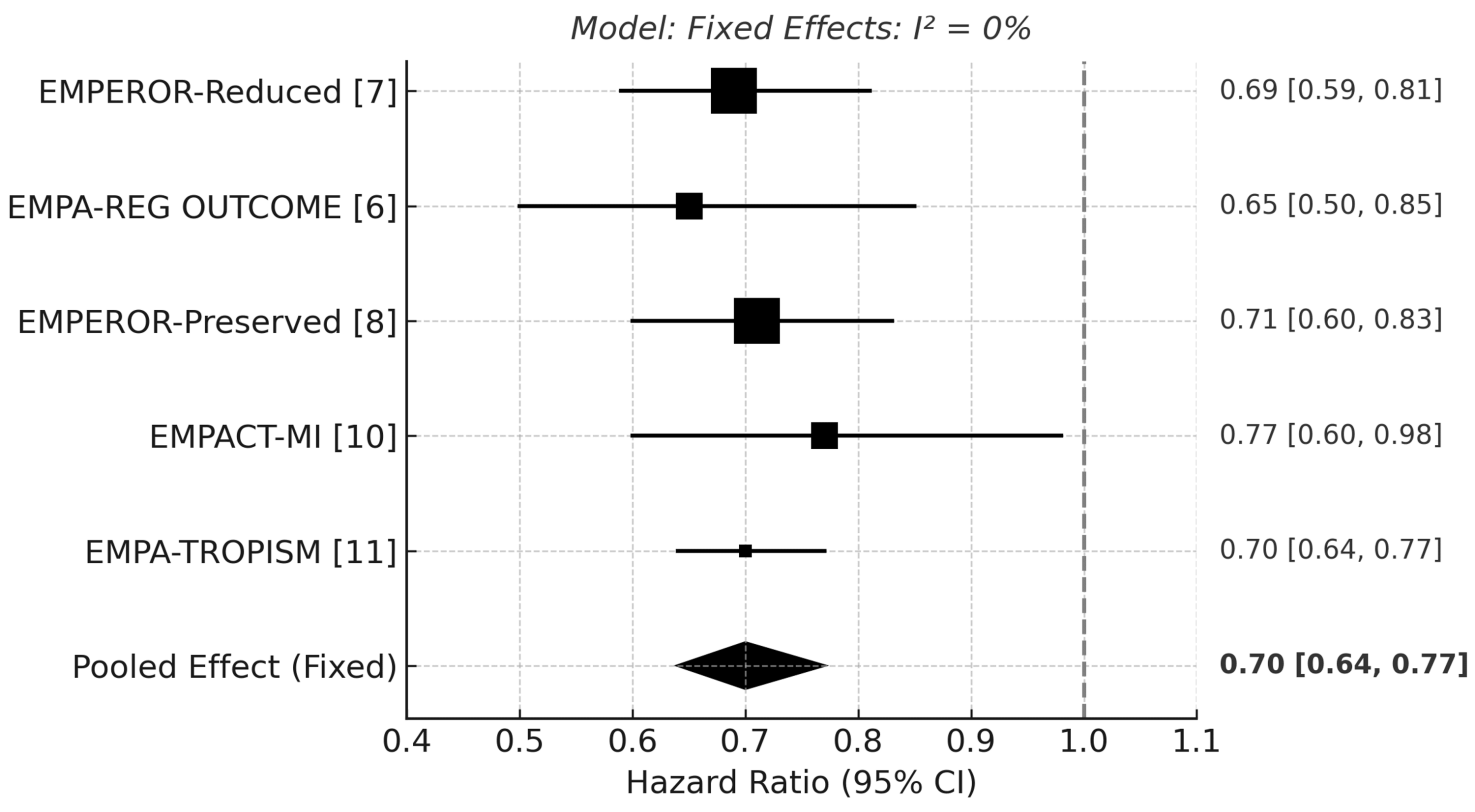
Forest plot of hospitalization for heart failure (sensitivity analysis) [6,7,8,10,11] EMPEROR-Reduced: Empagliflozin Outcome Trial in Patients With Chronic Heart Failure and a Reduced Ejection Fraction; EMPA-TROPISM: Effects of Empagliflozin on Cardiac Structure, Function, and Clinical Status in Patients With Heart Failure With Reduced Ejection Fraction Without Diabetes; EMPA-REG OUTCOME: Empagliflozin Cardiovascular Outcome Event Trial in Type 2 Diabetes Mellitus Patients; EMPEROR-Preserved: Empagliflozin Outcome Trial in Patients With Chronic Heart Failure With Preserved Ejection Fraction; EMPACT-MI: Empagliflozin in Patients With Acute Myocardial Infarction

EMPA-TROPISM, a smaller trial involving non-diabetic patients with HFrEF, was included in a sensitivity analysis using continuity correction due to zero events in the empagliflozin group. When this study was added to the meta-analysis, the pooled HR for cardiovascular mortality remained similar at 0.86 (95% CI: 0.78 to 0.95), with substantial heterogeneity (I²=66%). (Figure 4), and the pooled HR for HHF was 0.70 (95% CI: 0.64 to 0.77), with no heterogeneity (I²=0%). (Figure 5)

These findings demonstrate that the inclusion of EMPA-TROPISM did not materially alter the overall results, affirming the robustness of the primary analysis.

Discussion

This systematic review and meta-analysis studied the effects of empagliflozin on cardiovascular outcomes using data from RCTs. Our findings align with previous research, confirming the cardiovascular benefits of empagliflozin [12]. These findings are consistent with those from major studies such as the EMPA-REG OUTCOME trial [6] and the EMPEROR-Reduced trial [7].

One of the key reasons behind empagliflozin’s positive cardiovascular effects is its impact on the body's blood flow and fluid balance, independent of its effects on blood sugar levels [13]. The drug increases urine production and sodium removal from the body, which helps decrease the workload on the heart by lowering both the preload and afterload [14]. These effects are especially helpful for patients with heart failure [15]. Another proposed mechanism involves empagliflozin’s ability to improve vascular function and reduce arterial stiffness, contributing further to its heart-protective effects [16]. Furthermore, the EMPA-REG OUTCOME trial reported reductions in arterial stiffness and improvements in vascular function, suggesting enhanced endothelial health [6]. The EMPEROR-Reduced trial found that empagliflozin promoted natriuresis and osmotic diuresis, resulting in reduced preload and afterload, thereby improving cardiac workload [7]. These hemodynamic effects are believed to contribute substantially to its cardiovascular benefits.

Our review also highlights that empagliflozin provides cardiovascular benefits even for patients who do not have diabetes. Traditionally, empagliflozin was primarily used to manage blood sugar levels in diabetic patients [17]. However, recent studies have shown that empagliflozin can protect the heart and reduce cardiovascular risks in non-diabetic patients as well, making it useful for a broader range of patients [18]. Notably, the EMPA-TROPISM trial, which included only non-diabetic patients with HFrEF, demonstrated significant improvements in left ventricular remodeling, peak VO₂, and N-terminal pro B-type natriuretic peptide (NT-proBNP) levels compared to placebo [9]. This supports the hypothesis that empagliflozin’s benefits extend beyond glycemic control and into direct cardiovascular effects, particularly in heart failure. This is important because it means empagliflozin could potentially benefit many more patients who have heart issues but do not necessarily suffer from diabetes [19]. One of the most consistent findings was a significant reduction in heart failure-related hospitalizations. This result is especially important because treating heart failure, particularly when patients have HFpEF, has historically been challenging [20]. HFpEF patients currently have limited treatment options, and empagliflozin offers a promising new therapeutic approach [21].

Despite these positive findings, some variability in results was observed, particularly regarding non-fatal heart attacks. The lack of significant reduction in non-fatal myocardial infarctions may be due to empagliflozin’s limited direct anti-atherosclerotic action, as opposed to its stronger effects on hemodynamic and heart failure-related outcomes. Additionally, the relatively short follow-up durations may not capture long-term plaque stabilization effects. Differences in the characteristics of participants, such as varying degrees of kidney health and severity of heart failure across the trials, might explain these inconsistent results. The EMPEROR-Preserved trial found that these differences in patient populations could significantly affect how patients respond to treatment [8]. The substantial heterogeneity observed in the cardiovascular mortality analysis (I²=73%) likely reflects clinical and methodological differences across the included trials. These differences include variations in patient populations (such as heart failure phenotype and diabetic status), trial designs, follow-up durations, and outcome definitions. Notably, the EMPA-REG OUTCOME trial contributed the largest sample size and showed a significant treatment effect, which likely influenced the overall pooled result. Although a sensitivity analysis excluding this trial was not conducted, the variability among studies should be considered when interpreting the pooled HR. Additionally, while the cardiovascular mortality outcomes were adjudicated within each trial, variations in definitions and adjudication procedures may exist and could contribute to heterogeneity. Interestingly, inclusion of EMPA-TROPISM reduced heterogeneity (I² from 73% to 66%) despite its small sample size and zero-event arm. This may reflect consistency in treatment effect direction rather than statistical weight, as EMPA-TROPISM aligned with the overall trend favoring intervention. This heterogeneity likely stems from differences in study populations (e.g., type 2 diabetes vs. non-diabetic, HFpEF vs. HFrEF), trial design (blinding and follow-up duration), and variations in how cardiovascular mortality was defined and adjudicated. Despite these differences, all trials used independent adjudication committees to enhance outcome validity. These findings are consistent with results seen for dapagliflozin, another SGLT2 inhibitor, which also demonstrated reduced heart failure hospitalizations and cardiovascular benefits in trials like DAPA-HF and DELIVER, supporting a potential class effect [22,23].

Previous large-scale studies also suggest the drug poses minimal risk for serious side effects such as ketoacidosis or kidney damage, making it safe for long-term use [24]. Specifically, large-scale studies involving thousands of participants have consistently reported low incidence rates of these serious side effects [6-8]. The EMPA-REG OUTCOME trial, for instance, observed no significant increase in ketoacidosis or acute kidney injury compared to placebo, even among patients with pre-existing kidney disease [6]. Similar safety results were found in other major trials such as EMPEROR-Reduced and EMPEROR-Preserved, confirming the drug’s strong safety record across diverse patient groups [8].

Limitations

Although this review was thorough, there were limitations. Firstly, the availability of detailed patient-level data was limited, restricting deeper subgroup analyses, which could have provided insights into specific populations who might benefit most from empagliflozin. Secondly, potential publication bias may have influenced our findings. Additionally, there were inconsistencies in how trial outcomes were reported across different studies, potentially affecting the comparability of results. Moreover, the duration of follow-up varied among studies, limiting our ability to fully assess long-term effects and safety profiles. Lastly, the heterogeneity in patient demographics and baseline characteristics across included studies might have contributed to variability in outcomes, making it difficult to generalize our conclusions broadly. Also, the EMPACT-MI trial, while promising, had a moderate risk of bias due to its open-label design and relatively short follow-up period of six months, limiting the strength of its long-term cardiovascular findings [10]. Future studies should explore in greater detail how exactly empagliflozin protects the heart. Longer-term trials looking at broader outcomes, including stroke, kidney function, and patient quality of life, would also be valuable. More detailed subgroup analyses could help determine which patient groups might benefit the most from empagliflozin treatment. Among future directions, long-term follow-up studies assessing hard endpoints like stroke, mortality, and renal decline are urgently needed. Additionally, quality-of-life assessments and studies in underrepresented groups, such as patients with preserved ejection fraction without diabetes, should be prioritized to optimize individualized therapy.

## Conclusions

This review confirms that empagliflozin improves cardiovascular outcomes, notably by reducing heart failure hospitalizations. While the pooled analysis suggests a significant reduction in cardiovascular mortality, it is important to note that this outcome was primarily driven by EMPA-REG OUTCOME, with other trials showing varying results. Current evidence most robustly supports the use of empagliflozin in patients with heart failure, both with reduced and preserved ejection fraction and/or type 2 diabetes. Although some studies suggest a favorable safety profile, we chose not to include safety outcomes in this review due to variability in reporting and risk of bias in certain trials. Future research should prioritize long-term follow-up and assess empagliflozin’s effects in more diverse populations, including those with preserved ejection fraction without diabetes or with chronic kidney disease, to guide broader clinical application and better understand the mechanisms underlying its cardioprotective effects.
